# Graphdiyne-Based All-Solid-State Passively Q-Switched Tm:YAP Laser at 2 μm

**DOI:** 10.3390/nano13152171

**Published:** 2023-07-26

**Authors:** Qing Wu, Yanyu Wang, Gang Zhao, Haibin Wu, Yi Hu, Mengke Wang

**Affiliations:** 1 Heilongjiang Province Key Laboratory of Laser Spectroscopy Technology and Application, Harbin University of Science and Technology, Harbin 150080, China; wuqing@buaa.edu.cn (Q.W.); wyy30424@163.com (Y.W.); gunther9527@outlook.com (G.Z.); woo@hrbust.edu.cn (H.W.); 2 School of Chemistry and Chemical Engineering, Nantong University, Nantong 226019, China; 2107320023@stmail.ntu.edu.cn

**Keywords:** graphdiyne, saturable absorber, passively Q-switched, all-solid-state laser

## Abstract

All-solid-state Tm lasers have a wider wavelength range and higher output power compared to other types of lasers. In this work, we demonstrate an all-solid-state, high repetition, Tm:YAP laser Q-switched by a graphdiyne (GDY) saturable absorber. The high-quality optical nonlinear material GDY, synthesized by a cross-coupling method, exhibits a strong nonlinear optical response. The application of GDY as a saturable absorber in the passively Q-switched (PQS) Tm:YAP of an all-solid-state laser has been realized with the shortest pulse duration of ~785 ns and repetition frequency of ~199.6 kHz at a central wavelength of 1985.8 nm. This represents the shortest pulse duration and the highest repetition frequency achieved from GDY in a solid-state Tm laser to date. Our work demonstrates the remarkable nonlinear optical properties of GDY, which holds promising potential in the field of optoelectronics.

## 1. Introduction

All-solid-state lasers, with the advantages of small size, lightweight, compact structure, and long life, are widely used in military, medical, scientific research, and other fields. High-repetition frequency, short pulse duration solid-state lasers are widely used in many fields such as lidar, laser ranging, laser precision processing, and optical communication. The 2 μm is in the atmospheric window and human-eye-safe band, and there are many absorption peaks of atoms and molecules in the 2 μm, which makes the Tm:YAP crystal, an ultrafast laser crystal at 2 μm with good thermal and mechanical properties, widely used in the field of solid-state lasers. All-solid-state lasers can obtain high-repetition frequency narrow pulse width laser output by actively Q-switched and PQS methods. The PQS makes it easier for the short pulse duration laser output because no additional polarizing optics are required.

The saturable absorbers play a very important role as a nonlinear optical modulator in PQS pulsed solid-state lasers, taking advantage of the fact that the transmittance of a saturable absorber increases with the intensity of the input laser, allowing for the transition from continuous to pulsed lasers. The semiconductor saturable absorber mirror (SESAM) has developed rapidly as a saturable absorber over the past few decades and has been used in fiber lasers, solid-state lasers, and wafer lasers [1]. However, the development of SESAM is limited by its low damage threshold, high cost, and complex preparation process [2]. Subsequently, two-dimensional (2D) material such as graphene [3], topological insulators (TIs) [4,5], transition metal dichalcogenides (TMDs) [6,7,8,9], black phosphorus (BP) [10,11,12], MXene [13,14,15], perovskite [16], and other 2D materials are applied to the field of ultrafast lasers. Graphene has broadband optical modulation properties, but low absorption efficiency and poor modulation depth at 2 μm [17]. The preparation of TIs is complex, and the properties of TMDs are unstable [18]. The absorption peak of MXene is usually concentrated in the near-infrared band, and the absorption intensity is low [19]. BP-based saturable absorbers are prone to oxidation in the air [20]. Saturable absorbers with low optical losses, short pulse widths, strong nonlinearity, and fast response times need to be explored to obtain excellent pulsed lasers in a solid-state laser.

As a new member of the carbon family, GDY is a new all-carbon nanostructured material developed and synthesized after graphene, which has the characteristics of both 2D layered planar materials like graphene and three-dimensional porous material. The GDY molecular structure belongs to the π-electron conjugated system, where carbon atoms are interconnected by covalent bonds to form a planar structure. This structure imparts GDY with a high polarization rate as well as a nonlinear polarization rate. Using density-functional theory calculations, several light metals adsorb GDY structures denoted as AM3@GDY (AM = Li, Na, K). This structure has an intramolecular electron donor-acceptor framework, which endows them with a rare nonlinear optical behavior [21]. First-principles calculations show that GDY has a natural band gap compared to graphene with zero band gap, and the minimum band gap of GDY is between 0.46 eV and 1.22 eV depending on the calculation method and correlation function [22]. Furthermore, it is worth noting that the band gap of GDY is tunable. This means that the energy gap of materials can be effectively adjusted, leading to changes in the probability of electron transitions within GDY. This tunability can be achieved through various methods, such as chemical doping or stress modulation. GDY possesses unique characteristics such as a direct band gap semiconductor with a highly conjugated 2D π-electron system. GDY with these properties has become a significant research material in the field of optical nonlinearity. In 2010, Li et al. from the Chinese Academy of Sciences made a significant breakthrough in the synthesis of GDY. They successfully synthesized large-area thin films of GDY by coupling the reaction of hexaethylbenzene under the catalytic action of copper sheets [23]. This achievement marks a pivotal moment in the transition of GDY from a purely theoretical structure to an experimental platform. GDY is a narrow band gap semiconductor with a lower electron-hole recombination rate and a faster optical response speed, thus possessing stronger nonlinear optical properties. Based on the strong third-order nonlinear effect of GDY, the shortest output pulse width in an erbium-doped GDY saturable absorber fiber laser is 135.8 fs to date [24]. In the field of solid-state lasers, GDY saturable absorber is used in PQS Yb:SSO lasers to achieve a pulse output with a repetition rate of 43.6 kHz, a pulse width of 4.153 μs, and an average output power of 0.393 W. Improving the modulation parameters of the saturable absorber is necessary to realize a higher-performance pulsed laser. The application of GDY in fiber mode-locked lasers has gradually matured, while research in the field of solid-state lasers is still in its infancy.

In this article, we report, for the first time, an all-solid-state PQS Tm:YAP laser integrating a GDY-based saturable absorber fabricated from cross-coupling-synthesized GDY and test its third-order nonlinear optical properties The PQS Tm:YAP laser based on a GDY saturable absorber achieves the shortest pulse width of 785 ns, the corresponding repetition of 199.6 kHz at the central wavelength of 1985.8 nm. Compared with the reported results [25,26,27], the proposed GDY-based saturable absorber all-solid-state PQS in this work has the advantages of a stable resonator, large repetition rate, and narrow pulse width. It is expected that our findings will shed new light on the study of 2D GDY materials in ultrafine photonics and have great significance for the application of GDY and the development of novel nonlinear optical materials.

## 2. Preparation and Characterization of Saturable Absorber Devices

### 2.1. Materials

Two-dimensional GDY NSs are successfully synthesized by a cross-coupling method using hex ethynyl benzene (HEB) as a monomer and Cu foil as a substrate and catalyst, as previously reported [23,28]. The synthesized GDY is first dispersed in IPA solvent, and the GDY NSs are obtained by a facile liquid phase exfoliation in a water bath with a fixed temperature of 10 °C. The thicker GDY nanostructures are removed using centrifugation at 7500 rpm for 20 min, and the supernatant containing thinner GDY NSs is gently decanted into another tube, which is further centrifugated at 18,000 rpm for 30 min. The precipitate is collected and dried under a vacuum at 70 °C overnight.

**Figure 1:**
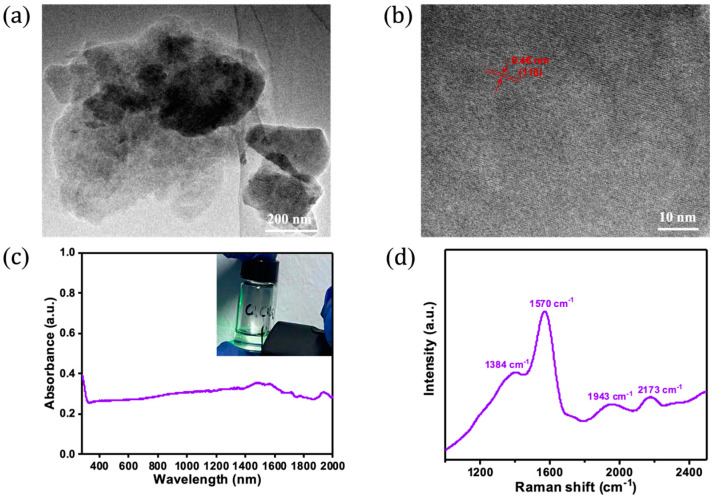
Structure characterization of GDY NSs. (**a**) TEM image; (**b**) HRTEM image; and (**c**) UV-vis-NIR spectrum of 2D GDY; inset in (**c**) shows the dispersion containing 2D GDY; (**d**) Raman spectrum.

The morphology of the as-fabricated GDY NSs is studied by FEI Talos200F transmission electron microscopy (TEM, 200 kV, FEI, Hillsboro, OL, USA). The atom arrangement of the as-fabricated GDY NSs is studied by high-resolution TEM (HRTEM). The optical absorption spectrum is performed through the UV-3150 UV-vis-NIR spectrophotometer (Hitachi S4000, Santa Clara, CA, USA). The TEM image shows that the as-fabricated 2D GDY NSs have a lateral size of 300–1200 nm (Figure 1a). The HRTEM image in Figure 1b shows that a clear lattice space (red line) of 0.46 nm can be observed, which can be assigned to the (110) plane GDY [29]. The UV-Vis-NIR spectrum exhibits that the 2D GDY NSs have a broadband absorption range from 300 nm to 2000 nm (Figure 1c), and the typical Tyndall can also be observed (Figure 1c inset), which holds great promise in ultrafast lasers. Figure 1d shows the typical Raman spectrum of the as-fabricated GDY NSs with four characteristic peaks at 1384, 1570, 1943, and 2173 cm^−1^, in good agreement with the previously reported result [29,30].

### 2.2. Devices

The GDY saturable absorber is prepared via the liquid-phase stripping approach. The 10 mg GDY powder is dissolved in 20 mL of alcohol solution (concentration 99.9%) and sonicate for 30 min to obtain a homogeneous dispersion solution with a concentration of 0.5 mg/mL and placed in a brown reagent bottle to avoid changes in the chemical properties of GDY caused by light. The prepared mixed solution is put into the ultrasonic grinder (ZOLLO, JY92-IIN, China) four ultrasonic times, each time for 20 min, to form a solution of GDY NSs with fewer layers. Then, the CaF_2_ substrate with good light transmission performance and less energy absorption is placed in the center of the base of the centrifuge (KW-4A). After sonication, an appropriate amount of the upper mixed solution is extracted and slowly added dropwise to the CaF_2_ substrate. The substrates are rotated at 2000 rpm for 30 s and then dried at ambient temperature (25 °C) for 10 h, resulting in good transparency of the prepared saturable absorber.

**Figure 2:**
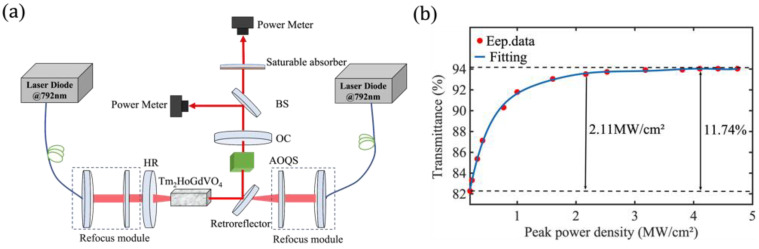
The nonlinear performance of the GDY. (**a**) The measuring setup of an optical nonlinear; (**b**) the nonlinear transmittance curve of the GDY.

A Tm,Ho:GdVO_4_ acousto-optic Q-switched(AOQS) laser, in which two high-power laser diodes are used as the pump sources, is constructed to measure the nonlinear absorption of the GDY saturable absorber, and the device diagram is shown in Figure 2a. The pump laser is focused through a collimated focusing system onto a Tm,Ho:GdVO_4_ crystal, where the pump source is first collimated using a collimating mirror with a focal length of 25 mm, and then passes through a focusing mirror with a focal length of 50 mm. The left-side laser passes through a high reflective (HR) planar-concave mirror, which has a radius of curvature of 150 mm and is coated on both sides with a highly transmissive film of 790–810 nm, and on the concave side, a highly reflective film of 1.9–2.1 µm. The laser on the right side passes through a 45° plate reflector, where the front and back sides are coated in high transmittance 790–810 nm and high reflectance 1.9–2.1 μm film. After that, the laser is transmitted from the crystal, reflected into the AOQS, and finally passes through the output coupler (OC), which is a flat reflector with a 20% light transmission film of 1.9–2.1 µm coated on the inside of the mirror. The laser beam output from the Tm,Ho:GdVO_4_ crystal is split into two beams using a beam-splitter mirror. One beam of light is directed toward a power meter, serving as the reference beam, while the other beam of light passes through GDY and is received by another power meter. By comparing the output power of the reference beam with that of the measurement beam, the magnitude of the nonlinear absorption can be determined. The measured data and the fitted curve are shown in Figure 2b [24]. The modulation depth, initial transmittance, and saturation absorption intensity of the GDY-based saturable absorber are 11.74%, 82.29%, and 2.11 MW/cm^2^, respectively.

## 3. Experimental Setup

**Figure 3:**
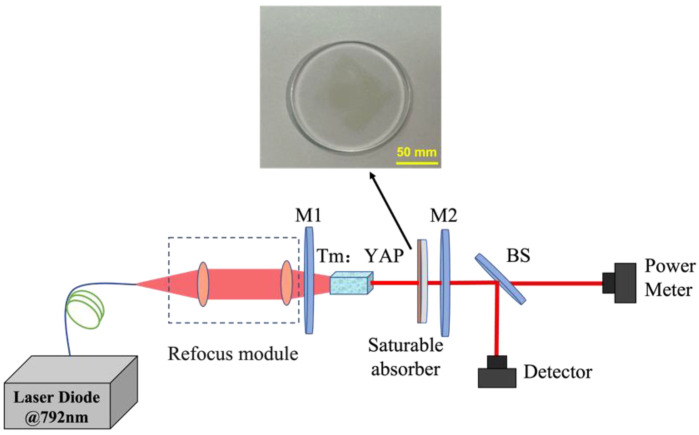
The experimental setup of a PQS Tm:YAP laser.

The experimental schematic diagram of an all-solid-state PQS Tm:YAP laser based on a GDY saturable absorber is illustrated in Figure 3. A fiber-coupled semiconductor laser, with a central output wavelength, core diameter, and numerical aperture of 792 nm, 105 μm, and 0.22, respectively, is used as a pump source. The collimated focusing system comprises a 25 mm focal length convex lens and a 50 mm convex lens. To start with, use a collimating lens with a focal length of 25 mm to accurately collimate the pump source. Then, employ a focusing lens with a focal length of 50 mm to achieve precise focus. The Tm:YAP crystal used has a Tm^3+^ concentration of 3%, dimensions of 3 mm × 3 mm × 8 mm, and an antireflection (AR) coating of 1.9–2.1 µm and 790–810 nm. The input coupler, M1 (curvature radius of 100 mm), is a flat concave mirror coated with a highly transmissive film at wavelengths of 790–810 nm and a highly reflective film at 1.9–2.1 µm. The pump beam is focused to the end face of the Tm:YAP crystal after passing through M1, forming a focused spot of 210 μm. The output coupler, M2, is a plain mirror coated with a pair of high reflectors at wavelengths in the range of 1.9–2.1 μm, and two different output mirrors with the transmittance of 2% and 5% are used for comparison. The total length of the laser resonant cavity is 35 mm. Utilizing the ABCD beam transport matrix, the spot radii on HR, saturable absorber, and OC can be calculated to be 157 μm, 128.5 μm, and 127 μm, respectively. To enhance cryogenic cooling efficiency and to prevent premature thermal lensing that can impede laser beam output, the crystal is tightly enveloped by a thermally conductive silver foil layer before the experiment. The crystal is securely installed onto a copper metal heat sink, and the base of the heat sink is connected to the circulating water system of a water chiller, ensuring that the water temperature is maintained at a constant 13 °C. Moreover, it is crucial to maintain a moderate ambient temperature throughout the experiment to prevent the formation of water droplets on the cooled crystal’s surface.

## 4. Results and Discussion

### 4.1. Results

**Figure 4:**
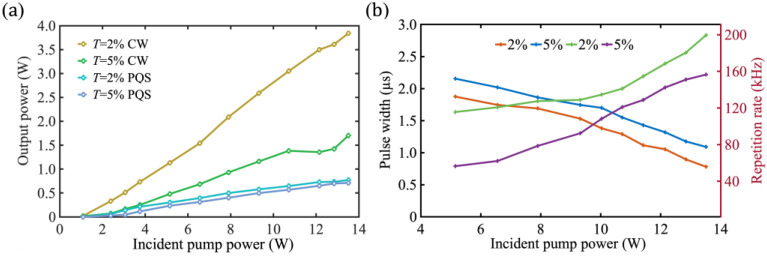
PQS Tm:YAP laser characteristics. (**a**) Output power; (**b**) pulse width and pulse repetition frequency.

The continuous waves (CW) and PQS laser output power as a function of incident pump power ranging from 1.09 to 13.51 W are demonstrated in Figure 4. There is a linear increase in the output power of the Tm:YAP laser as the incident pump power increases. The output power of the CW Tm:YAP laser is 3.84/1.70 W with T = 2/5% at 2 μm, corresponding to a slope efficiency of 28.4/12.6% at an incident pump power of 13.51 W. The maximum output power achieved using a T = 2% plane mirror as an output coupler in PQS mode is 0.77 W at an incident pump power of 13.51 W, corresponding to a slope efficiency of 5.7%. For an output coupler with T = 5%, the maximum output power and slope efficiency are 0.71 W and 5.3%, respectively. When comparing the output characteristics of the laser under CW operation and PQS mode, it is apparent that the laser threshold increases and the conversion efficiency decreases in PQS operation. The insertion of saturable absorber results in increased losses in the laser’s optical resonator, ultimately leading to a decrease in maximum output power.

**Figure 5:**
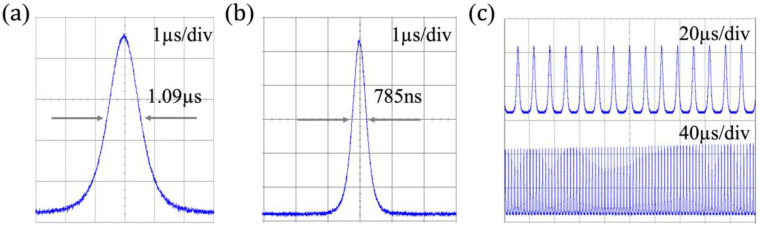
The shortest pulse shape and the typical pulse trains. (**a**) The temporal shortest pulse shape of T = 5%; (**b**) the temporal shortest pulse shape of T = 2%; (**c**) stable Q-switched pulse trains of T = 2% (20 μs/div and 40 μs/div).

The temporal pulse trains of the PQS Tm:YAP laser are detected using a detector (EOT, ET-5000, America) with bandwidth and displayed in an oscilloscope (Siglent, SDS5054X, bandwidth of 500 MHz, Shenzhen, China) with a sampling rate of 1 GSa/s. The relationship between the pulse width and repetition frequency curve of the output of the PQS Tm:YAP laser is shown in Figure 4b. The maximum repetition rate and the shortest pulse width are 199.6/156.5 kHz and 0.785/1.09 μs, respectively, at T = 2%/5%. The angle of the optical elements is finely adjusted to overcome noise interference from relaxation oscillations, after which the laser outputs a stable pulse as shown in Figure 5. The narrowest pulse monopoles measured at a time scale of 1 μs/div are shown in Figure 5a when T = 5%. Figure 5b,c show typical pulse trains recorded at T = 2% under different time scales, exhibiting the feature of the Q-switched laser.

**Figure 6:**
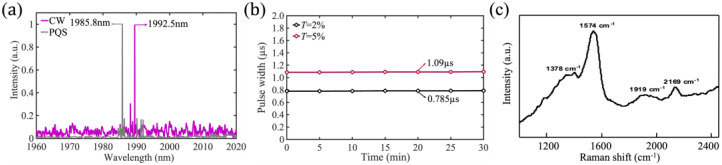
(**a**) The output wavelengths of the CW and PQS Tm:YAP laser; (**b**) stability of the laser output pulses; (**c**) Raman spectrum after laser experiments.

The output wavelength of the Tm:YAP laser is measured using a Bristol Instruments 721A-IR wavelength meter, as shown in Figure 6a. The CW Tm:YAP laser has a central wavelength output of 1992.5 nm, while the PQS Tm:YAP laser has a central wavelength output of 1985.8 nm when using a 2% transmittance output mirror. The central wavelength of the laser output in PQS mode is observed to be smaller than that of the laser output in CW mode. This difference is because the energy storage of the laser crystal emission section in PQS mode is greater than that in continuous operation mode, resulting in a decrease in crystal-stimulated emission cross-section and a spectral blue shift [31]. During the entire experimental process, the GDY-based PQS laser outputs a stable pulse over 30 min, and the variation of the width of the narrowest output laser pulse with time is shown in Figure 6b, evidenced by the stable structure of 2D GDY NSs characterized by the Raman technique with unchanged Raman signal after laser experiments (Figure 6c), which is sufficient to demonstrate the stability of the PQS laser.

Table 1 summarizes the output performance of PQS Tm:YAP lasers equipped with various material saturable absorbers. The saturable absorber based on GDY in this experiment shows exceptional output properties such as the shortest pulse width and the highest repetition rate when used as a modulator compared to similar and different materials.

### 4.2. Discussion

We have successfully prepared saturable absorber devices for Tm:YAP ultrafast laser using the ultrasonic liquid-phase stripping method on CaF_2_ substrates. The choice of CaF_2_ as the substrate material is based on its excellent transmittance and low absorption energy, which is ideal for this application. In addition, in past decades, the methods commonly used to prepare saturable absorber devices are the ultrasonic liquid-phase stripping method, the CVD method, and the sol-gel method. Among them, the slow deposition rate of the CVD method and the low safety of the reaction source and residual gas has limited its application, while the sol-gel method is difficult to prepare gel-like organic solvents with difficult rationing and a long solidification time to form a film. From the preparation process and economic point of view, the GDY saturable absorber prepared by ultrasonic liquid-phase stripping method is more advantageous.

In the experiment, a double-ended pumped Tm,Ho:GdVO_4_ AOQS laser with an output wavelength at 2 μm was constructed to investigate the nonlinear absorption characteristics of the GDY saturable absorber. By comparing the extensive document, the modulation depth of BP, Ti_3_C_2_T_x_, MoS_2_, Bi_2_Te_3_, and Nb_2_AlC are 5% [39], 11.1% [40], 2.9% [41], 9.8% [42], and 4.1% [43], respectively. The modulation depth of the GDY saturable absorber measured in this work is 11.74%. The GDY material exhibits a higher nonlinear optical response during optical transmission, enabling it to efficiently absorb and modulate light signals. Consequently, GDY applied in saturable absorber devices can achieve a greater modulation depth, leading to a more pronounced nonlinear optical effect.

GDY and its third-order nonlinear properties have found numerous applications in the field of fiber optics. However, research exploring the utilization of GDY in solid-state lasers has been relatively limited. In this paper, with the uniformity of GDY quantum dots and the excellent performance of the fabricated saturable absorber device, we have successfully achieved a stable Q-switched pulse output. Minimum pulse width and maximum pulse repetition frequency are obtained by compressing the resonant cavity length to optimize the laser performance. In addition, we adopted several measures to mitigate the effect of temperature on the experimental results. A water-cooled circulator was used in the experiments to maintain a stable temperature in the system and dissipate the heat generated during operation. We also carefully controlled the ambient temperature around the experimental setup to minimize fluctuations that could affect the laser’s performance.

## 5. Conclusions

In summary, we have, for the first time, demonstrated an all-solid-state, high repetition frequency PQS Tm:YAP laser with a 785 ns pulse duration and 199.6 kHz repetition frequency, Q-switched using a GDY saturable absorber. The laser design achieved the shortest pulses and highest repetition frequency with a short cavity length (~35 mm) to date. Our simple all-solid-state Tm:YAP laser design and GDY can promote the development of utilizing optical nonlinear materials and ultrafast laser crystals at 2 μm to generate the shortest pulses.

## Figures and Tables

**Table 1:** Comparison with the output performances of PQS laser with different materials.

Saturable Absorbers	Crystal	Central Wavelength (nm)	Average Output Power (mW)	Repetition Frequency (kHz)	Pulse Width (μs)	Year	Ref.
Graphene	Tm:YAP	1936.6	1370	98.6	2.164	2016	[32]
BP	Tm:YAP	1988.0	151	19.3	1.780	2016	[32]
MXene	Tm,Gd:CaF_2_	1906.0	1927	19.61	2.390	2019	[33]
SnSe_2_	Tm:YAP	1936.0	411	43.2	2.400	2020	[34]
WS_2_	Tm:YALO_3_	1936.0	110	34.7	2.650	2020	[35]
PZT	Tm:YAP	1991.9	810	175.0	1.690	2020	[36]
GDY	Tm:YAP	1936.6	1370	98.59	2.164	2020	[37]
GDY	Ho:YLF	2062.1	443	29.72	1.380	2021	[38]
GDY	Tm:YLF	1908.4	1290	91.58	2.207	2021	[25]
GDY	Tm:YAP	1985.8	770	199.6	0.785	2023	This work

## Data Availability

The data that support the findings of this study are available from the corresponding author upon reasonable request.

## References

[1] Hakobyan S., Wittwer V.J., Hasse K., Krankel C., Sudmeyer T., Calmano T. (2016). Highly efficient Q-switched Yb:YAG channel waveguide laser with 5.6 W of average output power. Opt. Lett..

[2] Li P.F., Chen Y., Yang T.S., Wang Z.Y., Lin H., Xu Y.H., Li L., Mu H.R., Shivananju B.N., Zhang Y.P. (2017). Two-Dimensional CH_3_NH_3_PbI_3_ Perovskite Nanosheets for Ultrafast Pulsed Fiber Lasers. ACS Appl. Mater. Interfaces.

[3] Novoselov K.S., Geim A.K., Morozov S.V., Jiang D., Zhang Y., Dubonos S.V., Grigorieva I.V., Firsov A.A. (2004). Electric field effect in atomically thin carbon films. Science.

[4] Yu Z.H., Song Y.R., Tian J.R., Dou Z.Y., Guoyu H.Y., Li K.X., Li H.W., Zhang X.P. (2014). High-repetition-rate Q-switched fiber laser with high quality topological insulator Bi_2_Se_3_ film. Opt. Express.

[5] Cheng P.Y., Du Y.Q., Han M.M., Shu X.W. (2020). Mode-locked and Q-switched mode-locked fiber laser based on a ferroferric-oxide nanoparticles saturable absorber. Opt. Express.

[6] Su X.C., Nie H.K., Wang Y.R., Li G.R., Yan B.Z., Zhang B.T., Yang K.J., He J.L. (2017). Few-layered ReS_2_ as saturable absorber for 2.8 μm solid state laser. Opt. Lett..

[7] Lin T., Sun H., Wang X., Mao D., Wang Y.G., Li L., Duan L.N. (2015). Passively Q-switched Nd:YAG laser with a MoS_2_ solution saturable absorber. Laser Phys..

[8] Lin J., Hu Y.Y., Chen C.J., Gu C., Xu L.X. (2015). Wavelength-tunable Yb-doped passively Q-switching fiber laser based on WS_2_ saturable absorber. Opt. Express.

[9] Cheng C., Liu H.L., Tan Y., de Aldana J.R.V., Chen F. (2016). Passively Q-switched waveguide lasers based on two-dimensional transition metal diselenide. Opt. Express.

[10] Zhang M., Wu Q., Zhang F., Chen L.L., Jin X.X., Hu Y.W., Zheng Z., Zhang H. (2019). 2D Black Phosphorus Saturable Absorbers for Ultrafast Photonics. Adv. Opt. Mater..

[11] Wang M.K., Zhu J., Zi Y., Wu Z.G., Hu H.G., Xie Z.J., Zhang Y., Hu L.P., Huang W.C. (2021). Functional two-dimensional black phosphorus nanostructures towards next-generation devices. J. Mater. Chem. A.

[12] Huang W.C., Li C., Gao L.F., Zhang Y., Wang Y.Z., Huang Z.Y.N., Chen T.T., Hu L.P., Zhang H. (2020). Emerging black phosphorus analogue nanomaterials for high-performance device applications. J. Mater. Chem. C.

[13] Wu Q., Wang Y.Z., Huang W.C., Wang C., Zheng Z., Zhang M., Zhang H. (2020). MXene-based high-performance all-optical modulators for actively Q-switched pulse generation. Photonics Res..

[14] Wang M.K., Zhu J., Zi Y., Huang W.C. (2021). 3D MXene Sponge: Facile Synthesis, Excellent Hydrophobicity, and High Photothermal Efficiency for Waste Oil Collection and Purification. ACS Appl. Mater. Interfaces.

[15] Huang W.C., Ma C.Y., Li C., Zhang Y., Hu L.P., Chen T.T., Tang Y.F., Ju J.F., Zhang H. (2020). Highly stable MXene (V2CTx)-based harmonic pulse generation. Nanophotonics.

[16] Niu Z.Q., Li G.Q., Yang K.J., Li T., Zhao J., Zhao S.Z., Li D.C., Qiao W.C., Chu H.W., Feng T.L. (2019). Doubly Q-switched Tm:YAP laser with g-C_3_N_4_ saturable absorber and AOM. Opt. Mater..

[17] Jin C.J., Bai Y., Li L.F., Jiang T., Ren Z.Y., Bai J.T. (2015). A single-frequency, graphene-based passively Q-switched Tm: YAP laser. Laser Phys..

[18] Sun Z.P. Optical Modulators with Two-dimensional Layered Materials. Proceedings of the Progress in Electromagnetic Research Symposium (PIERS).

[19] Zheng Z.X., Guo C.Y., Wang E.H., He Z.J., Liang T.X., Yang T., Hou X.M. (2021). The oxidation and thermal stability of two-dimensional transition metal carbides and/or carbonitrides (MXenes) and the improvement based on their surface state. Inorg. Chem. Front..

[20] Xu Y.P., Zhu X.H., Lu Z.B., Zhang G.G. (2021). Effects of oxygen atoms and oxygen molecules on the electronic properties of modified black phosphorus. Chem. Phys..

[21] Gao X., Liu H.B., Wang D., Zhang J. (2019). Graphdiyne: Synthesis, properties, and applications. Chem. Soc. Rev..

[22] Huang C.S., Li Y.J., Wang N., Xue Y.R., Zuo Z.C., Liu H.B., Li Y.L. (2018). Progress in Research into 2D Graphdiyne-Based Materials. Chem. Rev..

[23] Li G.X., Li Y.L., Liu H.B., Guo Y.B., Li Y.J., Zhu D.B. (2010). Architecture of graphdiyne nanoscale films. Chem. Commun..

[24] Wu Q., Chen S., Bao W.L., Wu H.B. (2022). Femtosecond Pulsed Fiber Laser Based on Graphdiyne-Modified Tapered Fiber. Nanomaterials.

[25] Zu Y.Q., Guo J., Hao Q.Q., Zhang F., Wang C., Liu J., Wang B. (2021). Graphdiyne as a saturable absorber for 2-μm all-solid-state Q-switched laser. Sci. China-Mater..

[26] Zong M.Y., Zu Y.Q., Guo J., Zhang Z., Liu J.J., Ge Y.Q., Liu J., Su L.B. (2021). Broadband nonlinear optical response of graphdiyne for mid-infrared solid-state lasers. Sci. China-Phys. Mech. Astron..

[27] Yang Q., Zhang X.Y., Yang Z.X., Ren X.H., Wang J., Li Q.D., Cui X.L., Zhu X.L. (2019). Broadband gamma-graphyne saturable absorber for Q-switched solid-state laser. Appl. Phys. Express.

[28] Matsuoka R., Sakamoto R., Hoshiko K., Sasaki S., Masunaga H., Nagashio K., Nishihara H. (2017). Crystalline Graphdiyne Nanosheets Produced at a Gas/Liquid or Liquid/Liquid Interface. J. Am. Chem. Soc..

[29] Zhou J.Y., Gao X., Liu R., Xie Z.Q., Yang J., Zhang S.Q., Zhang G.M., Liu H.B., Li Y.L., Zhang J. (2015). Synthesis of Graphdiyne Nanowalls Using Acetylenic Coupling Reaction. J. Am. Chem. Soc..

[30] Hu Y., Wang M.K., Hu L.P., Hu Y.L., Guo J., Xie Z.J., Wei S.R., Wang Y.H., Zi Y., Zhang H. (2022). Recent advances in two-dimensional graphdiyne for nanophotonic applications. Chem. Eng. J..

[31] Yan B.Z., Zhang B.T., He J.L., Nie H.K., Li G.R., Liu J.T., Shi B.N., Wang R.H., Yang K.J. (2019). Ternary chalcogenide Ta_2_NiS_5_ as a saturable absorber for a 1.9 μm passively Q-switched bulk laser. Opt. Lett..

[32] Chu Z.Z., Liu J., Guo Z.N., Zhang H. (2016). 2 μm passively Q-switched laser based on black phosphorus. Opt. Mater. Express.

[33] Zu Y.Q., Zhang C., Guo X.S., Liang W.Y., Liu J., Su L.B., Zhang H. (2019). A solid-state passively Q-switched Tm,Gd:CaF_2_ laser with a Ti_3_C_2_T_x_ MXene absorber near 2 μm. Laser Phys. Lett..

[34] Ran B.F., Sun H.Y., Ma Y.F. (2020). Two-dimensional tin diselenide passively Q-switched 2 μm Tm:YAP laser. Infrared Phys. Technol..

[35] Ma Y.F., Sun H.Y., Ran B.F., Zhang S.C., Zhang H., Tittel F.K., Lv Z.W. (2020). Passively Q-switched Tm:YAlO_3_ laser based on WS_2_/MoS_2_ two-dimensional nanosheets at 2 µm. Opt. Laser Technol..

[36] Li S.C., Li L.J., Qi T.Q., Wu Z.Y., Duan X.M., Yang X.N., Shen Y.J. (2021). Passively Q-switched Tm:YAP laser with a lead zirconate titanate saturable absorber. Appl. Opt..

[37] Liu W.X., Zu Y.Q., Guo J., Huang S.J., Li D.K., Liu J., Liu D.H., Ge Y.Q. (2021). Watt-level graphdiyne passively Q-switched Tm:YAP laser at similar to 2 μm. Optik.

[38] Zhang C., Hao Q.Q., Zu Y.Q., Zong M.Y., Guo J., Zhang F., Ge Y.Q., Liu J. (2020). Graphdiyne Saturable Absorber for Passively Q-Switched Ho^3+^-Doped Laser. Nanomaterials.

[39] Xie Y.X., Kong L.C., Qin Z.P., Xie G.Q., Zhang J. (2016). Black phosphorus-based saturable absorber for Q-switched Tm: YAG ceramic laser. Opt. Eng..

[40] Yuan J.H., Li J.R., Li L.J., Han J., Shen Y.J., Li Y.Y., Wu Z.Y., Li S.C. (2022). A Passively Q-switched Tm:YAlO_3_ bulk laser with a MXene Ti_3_C_2_T_x_ saturable absorber. Optik.

[41] Li L.J., Zhou L., Li T.X., Yang X.N., Xie W.Q., Duan X.M., Shen Y.J., Yang Y.Q., Yang W.L., Zhang H. (2020). Passive mode-locking operation of a diode-pumped Tm:YAG laser with a MoS_2_ saturable absorber. Opt. Laser Technol..

[42] Gao P., Huang H.Z., Wang X.H., Liu H.G., Huang J.H., Weng W., Dai S.T., Li J.H., Lin W.X. (2018). Passively Q-switched solid-state Tm:YAG laser using topological insulator Bi_2_Te_3_ as a saturable absorber. Appl. Opt..

[43] Hu Y.Y., Yang X.N., Li L.J., Gao Q., Li S.C., Wu Z.Y., Yang Y.Q., Cui H.Y., Zhou S. (2022). A passively Q-switched operation of Tm:YAP laser with a Nb_2_AlC-based saturable absorber. Optik.

